# LXR Agonist T0901317′s Hepatic Impact Overrules Its Atheroprotective Action in Macrophages, Driving Early Atherogenesis in Chow-Diet-Fed Male Apolipoprotein E Knockout Mice

**DOI:** 10.3390/biom14040429

**Published:** 2024-04-02

**Authors:** Menno Hoekstra, Laura M. de Jong, Rick van der Geest, Lidewij R. de Leeuw, Rani Krisnamurthi, Janine J. Geerling, Miranda Van Eck

**Affiliations:** 1 Division of BioTherapeutics, Leiden Academic Centre for Drug Research, Leiden University, 2333 CC Leiden, The Netherlands; l.m.de.jong@lacdr.leidenuniv.nl (L.M.d.J.); m.eck@lacdr.leidenuniv.nl (M.V.E.); 2 Division of Systems Pharmacology and Pharmacy, Leiden Academic Centre for Drug Research, Leiden University, Leiden, The Netherlands; 3 Pharmacy Leiden, Leiden, The Netherlands

**Keywords:** liver X receptor, atherosclerosis, lipid metabolism, liver, macrophage, cholesterol efflux, lipogenesis

## Abstract

Preclinical studies regarding the potential of liver X receptor (LXR) agonists to inhibit macrophage foam cell formation and the development of atherosclerotic lesions are generally executed in mice fed with Western-type diets enriched in cholesterol and fat. Here, we investigated whether LXR agonism remains anti-atherogenic under dietary conditions with a low basal hepatic lipogenesis rate. Hereto, atherosclerosis-susceptible male apolipoprotein E knockout mice were fed a low-fat diet with or without 10 mg/kg/day LXR agonist T0901317 supplementation for 8 weeks. Importantly, T0901317 significantly stimulated atherosclerosis susceptibility, despite an associated increase in the macrophage gene expression levels of cholesterol efflux transporters ABCA1 and ABCG1. The pro-atherogenic effect of T0901317 coincided with exacerbated hypercholesterolemia, hypertriglyceridemia, and a significant rise in hepatic triglyceride stores and macrophage numbers. Furthermore, T0901317-treated mice exhibited elevated plasma MCP-1 levels and monocytosis. In conclusion, these findings highlight that the pro-atherogenic hepatic effects of LXR agonism are dominant over the anti-atherogenic effects in macrophages in determining the overall atherosclerosis outcome under low-fat diet feeding conditions. A low-fat diet experimental setting, as compared to the commonly used high-fat-diet-based preclinical setup, thus appears more sensitive in uncovering the potential relevance of the off-target liver effects of novel anti-atherogenic therapeutic approaches that target macrophage LXR.

## 1. Introduction

The nuclear receptors liver X receptor alpha (LXR-alpha) and LXR-beta are multi-purpose players in the total body lipid metabolism. Within hepatocytes, LXR-alpha stimulates the activity of genes involved in lipogenesis, i.e., fatty acid and triglyceride synthesis [1,2,3]. As such, the treatment of *wild-type mice* with the synthetic LXR agonist T0901317 increases liver triglyceride stores and is associated with the development of hypertriglyceridemia [4,5]. LXRs also stimulate the removal of (excess) cholesterol from body through activating reverse cholesterol transport [6,7]. More specifically, they increase the expression of cholesterol efflux transporters ATP-binding cassette transporter A1 (ABCA1) and ABCG1 in macrophages [8,9] and stimulate hepatic expression levels of cholesterol 7alpha-hydroxylase (CYP7A1) and ABCG5/ABCG8, which convert cholesterol into bile acids and mediate the flux of cholesterol from hepatocytes into the bile, respectively [10,11]. The hypercholesterolemia-induced accumulation of cholesterol in macrophages, i.e., the generation of so-called foam cells, within the arterial wall is a key step in the development of atherosclerotic lesions, the primary underlying cause of cardiovascular diseases such as myocardial infarction and stroke [12]. Since reverse cholesterol transport protects macrophages from becoming foam cells [13], preclinical studies on genetically hypercholesterolemic mice have shown that treatment with synthetic LXR agonists is able to decrease atherosclerosis susceptibility [14,15,16,17].

Studies regarding the anti-atherogenic potential of LXR agonists are generally executed in mice fed with Western-type diets enriched in cholesterol and fat. Importantly, the contribution of hepatic lipogenesis to plasma lipid levels is probably minimal under these high-fat diet feeding conditions, considering the bulk influx of lipids from the intestine into the blood compartment. As such, the relative impact of LXR agonism-associated hypertriglyceridemia/dyslipidemia on the overall atherosclerosis outcome may, thus, far have been underestimated. To uncover whether LXR agonism remains anti-atherogenic under low-fat feeding conditions, in the current study, we evaluated the effect of chronic treatment with the pan LXR agonist T0901317 (EC50~50 nM for both LXR-alpha and LXR-beta subtypes [18]) on chow-diet-fed hypercholesterolemic atherosclerosis-susceptible apolipoprotein E (APOE) knockout mice.

## 2. Materials and Methods

### 2.1. Mice

Homozygous *APOE knockout mice* on a C57BL/6 background were originally obtained from The Jackson Laboratory and subsequently bred in-house at the Leiden Academic Centre for Drug Research, Gorlaeus Laboratories, Leiden, The Netherlands. The animal experiments were performed in temperature- and light cycle (12 h light/12 h dark)-controlled rooms. The protocols were approved by the Animal Ethics Committee of Leiden University/Leiden University Medical Center under project license number 14075 (issue date: 1 August 2014). The two experiments were executed according to the principles of laboratory animal care and regulations of Dutch law on animal welfare, the Directive 2010/63/EU of the European Union, and the ARRIVE guidelines.

Experiment 1: Twenty-two male *APOE knockout mice* of eight to ten weeks old were randomly assigned to two different experimental groups, while matching them for age and weight. The two groups of mice were group-housed with 3–5 mice per cage and provided with feeding containers within their cages that contained either a powder-based low-fat control chow diet or a powder form of the same chow diet supplemented with T0901317 (Apexbio Technology LLC/Gentaur Nederland BV, Eersel, The Netherlands; cat.nr. A2249) at the commonly used dose of 10 mg/kg/day [4,14,17]. Given that Chen et al. [19] previously showed that the effect of T0901317 treatment on hepatic lipid levels and atherosclerosis susceptibility is not gender-dependent in Western-type-diet-fed *APOE knockout mice*, the choice for the gender in our study was purely driven by the in-house availability of male mice. Group sizes of n = 11 were used, based upon a power calculation with the following input parameters: measurable effect: 25%, sigma: 20%, alpha: 0.05, power: 0.8 (https://www.stat.ubc.ca/~rollin/stats/ssize/n2.html; accessed on 1 August 2014). The mice were weighed once a week throughout the experiment and monitored daily for their overall well-being. Although we did quantify their food intake, an addition of T0901317 to the chow diet was not associated with a clear change in the amount of food consumed by the different groups of mice. Visual inspection of the food containers showed that similar amounts of food were left in the food containers during the weekly cage changes. After 8 weeks of diet feeding, the mice were killed through a subcutaneous injection with a terminal mixture of ketamine (100 mg/kg), xylazine (12.5 mg/kg), and atropine (125 µg/kg) and exsanguination by orbital bleeding into EDTA-coated tubes in the ad libitum fed, physiologically relevant metabolic state. Subsequently, the peritoneal cavity of the mice was lavaged with 10 mL of cold PBS to collect peritoneal leukocytes. Then, the arterial tree was perfused in situ with PBS (with the pressure of 100 mm Hg) for 10 min via a cannula in the left ventricular apex. Aortic arches containing the major branches towards the brachiocephalic artery, the left common carotid artery, and the left subclavian artery were cut loose from the heart and stored at −20 °C for biochemical analysis. Subsequently, the heart and aortic root were excised and stored in 3.7% neutral-buffered formalin for histochemical analysis. Livers were weighed and fixed overnight in 3.7% neutral-buffered formalin for histological analysis or stored at −20 °C for further biochemical analysis.

Experiment 2: Twelve male *APOE knockout mice* of eight to ten weeks old were randomly assigned to two different experimental groups, while matching them for age, plasma total cholesterol levels, and weight. The two groups of mice were housed with 1 to 3 mice per cage and provided with feeding containers within their cages that contained either a powder-based low-fat control chow diet or a powder form of the same chow diet supplemented with 10 mg/kg/day T0901317. Group sizes of n = 6 were used, based upon a power calculation with the following input parameters: measurable effect: 50%, sigma: 30%, alpha: 0.05, power: 0.8. After 4 days of diet feeding, the mice were killed through a subcutaneous injection with a terminal mixture of ketamine (100 mg/kg), xylazine (12.5 mg/kg), and atropine (125 µg/kg) and exsanguination by orbital bleeding into EDTA-coated tubes in the ad libitum fed, physiologically relevant metabolic state. Livers were weighed and stored at −20 °C for further biochemical analysis.

### 2.2. Hepatic Lipid Extraction and Quantification

Tissue lipids were extracted from ~50 mg of liver per mouse. For triglyceride extraction, the tissue pieces were homogenized in 500 µL of 5% Nonidet™ P 40 Substitute. The solution was subjected to two cycles of heating to 90 °C and cooling on ice for 2 min each. Subsequently, insoluble material was removed from the solution through centrifugation and triglycerides in the supernatant were measured using an enzymatic colorimetric assay (Roche Diagnostics, Mannheim, Germany). Cholesterol was extracted from similar-sized liver pieces using the protocol of Bligh and Dyer [20] and quantified using an enzymatic colorimetric assay [21]. The concentration of triglycerides and cholesterol in the liver samples is expressed as µg lipid/mg protein.

### 2.3. Gene Expression Analysis

A quantitative gene expression analysis was performed essentially as previously described [22]. The total RNA was isolated according to Chomczynski and Sacchi [23] and reverse transcribed using RevertAid^TM^ reverse transcriptase (Thermo Fisher Scientific, Bleiswijk, Netherlands). A gene expression analysis was performed using real-time SYBR Green technology (Eurogentec, Seraing, Belgium). Primer sequences can be provided on request. Beta-actin (ACTB), peptidylprolyl isomerase A/cyclophilin A (PPIA), glyceraldehyde-3-phosphate dehydrogenase (GAPDH), ribosomal protein L27 (RPL27), and acidic ribosomal phosphoprotein P0 (36B4) were used as the standard housekeeping genes. The relative gene expression numbers were calculated by subtracting the threshold cycle number (Ct) of the target gene from the average Ct of the housekeeping genes (Ct housekeeping) and raising 2 to the power of this difference.

### 2.4. Plasma Lipid and Cytokine Analyses

Plasma free cholesterol, cholesteryl ester, and triglyceride levels were determined in the plasma from all individual mice using enzymatic colorimetric assays (Roche Diagnostics and [21]). To generate the lipoprotein distribution profiles of the different groups of mice, the remaining plasma from the different mice was pooled per experimental group and subjected to fractionation using a Superose 6 column (injection: 30 µL pooled plasma + 20 µL PBS; 3.2 × 300 mm, Smart-system, Pharmacia, Sweden). Subsequently, the total cholesterol levels were measured in the different lipoprotein fractions using the same enzymatic colorimetric assay as that used for the original plasma samples, taking into account the efficacy of the fast-performance liquid chromatography column. Murine monocyte chemoattractant protein 1 (MCP-1/CCL2) protein levels were assayed in plasma specimens using an MCP-1 instant ELISA kit (eBioscience, Hatfield, UK), according to the manufacturer’s instructions.

### 2.5. Peritoneal Leukocyte Analysis

Peritoneal exudates were centrifuged at 1500 rpm for 10 min. Cell pellets were resuspended in 1 mL of cell DMEM culture medium containing 10% fetal bovine serum, penicillin/streptomycin, and L-glutamine and subjected to hematological analysis (100 µL volume; Sysmex XT-2000iV Veterinary Heamatology analyzer; Sysmex Corporation, Etten-Leur, The Netherlands) to measure the total leukocyte count. Manual re-gating was performed to identify which fraction of the peritoneal leukocytes was composed of monocytes, macrophages, or SSC^high^/FSC^high^ lipid-laden foam cells, as previously described [24]. A part of the remaining cell suspension (500 µL) was transferred to 24-well cell culture plates and left to incubate for 24 h at 37 °C in. Subsequently, the medium was collected for the measurement of the protein levels of the pro-inflammatory cytokines MCP-1 and interleukin-6 (IL-6), using, respectively, an MCP-1 instant ELISA kit (eBioscience, Hatfield, UK) or an ELISA MAX™ Deluxe Mouse IL-6 kit (Biolegend, San Diego, CA, USA), according to the manufacturer’s instructions. The residual peritoneal cells (~400 µL cell suspension) were pelleted by centrifugation at 1500 rpm for 10 min and stored at −20 °C in 500 µL of a guanidinium thiocyanate solution for subsequent gene expression analysis.

### 2.6. Blood Cell Analysis

The total leukocyte counts and the distribution over different subclasses of white blood cells, i.e., neutrophils, monocytes, and lymphocytes, were routinely measured in orbital blood specimens using an automated SYSMEX XT-2000iV Veterinary Haematology analyzer (SYSMEX Corporation).

### 2.7. Histological Analysis

Liver cryosections (7 µm) and cryostat sections of the aortic root (10 µm) were routinely stained with Oil red O to visualize neutral lipids and counterstained with hematoxylin. Oil red O-stained atherosclerotic lesion areas were quantified using a Leica DMRE microscope coupled to a video camera and Leica Qwin Imaging software 2.0 (Leica Ltd., Cambridge, UK). The mean lesion area (μm^2^) was calculated from 5 Oil red O-stained sections, starting at the appearance of aortic valves. Paraffin sections of the liver and cryosections of the aortic root were also stained immunohistochemically for the presence of macrophages using a rat anti-CD68 antibody (1:1000 dilution; AB53444; Abcam, Cambridge, UK). Goat anti-rat coupled to horse radish peroxidase (1:100 dilution; DAKO, Heverlee, Belgium) was used as a secondary antibody and BCIP/NBI substrate (DAKO, Heverlee, Belgium) was used for visualization and subsequent macrophage plaque area and liver macrophage number quantification. The atherosclerotic lesion collagen content was determined using Masson’s Trichrome staining (Sigma-Aldrich, Zwijndrecht, The Netherlands). All quantifications were performed by computer-aided morphometric analysis using the Leica image analysis system in a blinded manner.

### 2.8. Data Analysis

Data are presented as individual mouse values with the group means ± SEM. Statistical analysis was performed using Graphpad Instat software (San Diego, CA, USA, http://www.graphpad.com; accessed on 15 July 2023). Normality testing of the experimental groups was performed using the method Kolmogorov and Smirnov. A Grubb’s test was performed on the data to identify outliers. The significance of the differences between the two experimental groups was calculated using a *t*-test. A two-way analysis of variance (ANOVA) with a Bonferroni post-test was applied to uncover the potential impact of the time of diet feeding and dietary T0901317 supplementation on body weight. Probability values less than 0.05 were considered to be significant.

## 3. Results

**Figure 1:**
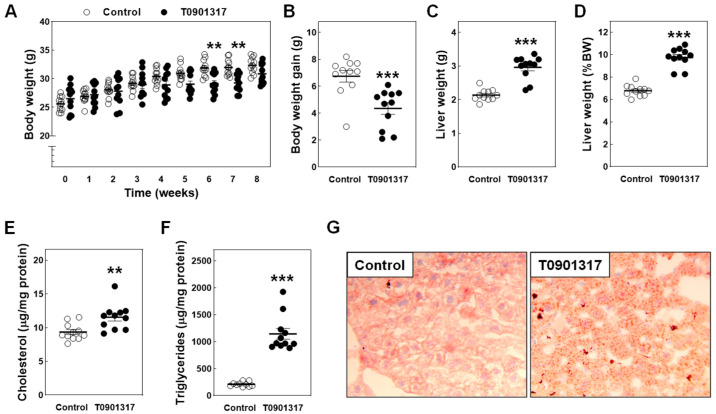
Body weight development (**A**), total body weight gain (**B**), absolute (**C**) and body-weight-corrected (**D**) liver weights, and hepatic cholesterol (**E**) and triglyceride (**F**) contents in male *APOE knockout mice* fed a control chow diet (white dots) or chow diet supplemented with 10 mg/kg/day LXR agonist T0901317 (black dots) for 8 weeks. Data represent the individual mouse values with the group means ± SEM. ** *p* < 0.01, *** *p* < 0.001 versus control. (**G**) Representative images of Oil red O-stained liver cryosections showing some lipid accumulation in control livers, but markedly increased neutral lipid deposition in T0901317-treated mice; Original magnification: 200×.

Atherosclerotic lesion development was initiated in chow-diet-fed *APOE knockout mice* at ~8 weeks of age [25]. To uncover whether the LXR agonist T0901317 was also able to diminish their susceptibility for the development of atherosclerotic lesions under low-fat diet feeding conditions, we therefore fed age-matched 8- to 10-week-old male *APOE knockout mice* with a control regular chow diet or a chow diet supplemented with 10 mg/kg/day T0901317 for 8 weeks. T0901317 treatment was associated with a significant decrease in total body weight gain (−36%; *p* < 0.01; Figure 1A,B), which fits with previous data from Dong et al. [26] and Gao et al. [27] that T0901317 impairs adipose tissue expansion and protects mice against obesity development. In contrast, both the absolute (+39%; *p* < 0.001; Figure 1C) and body-weight-corrected (+44%; *p* < 0.001; Figure 1D) liver weights were significantly elevated in the T0901317-treated mice as compared to the control mice.

The quantification of liver lipid stores indicated that the hepatomegaly was paralleled by a small but significant increase in hepatic cholesterol levels (+23%; *p* < 0.01; Figure 1E). In accordance with the notion that LXR agonism efficiently stimulates fatty acid and triglyceride synthesis, a marked 4.5-fold increase (*p* < 0.001) in hepatic triglyceride levels was also detected in response to T0901317 treatment (Figure 1F). Oil red O staining was used on cryosections of the livers to visually examine the level of hepatic neutral lipid accumulation. As can be seen from Figure 1G, some small-sized lipid stores were already present in the livers of regular chow-diet-fed *APOE knockout mice* at 16–18 weeks of age. This observation corroborates previous findings of Li et al. that APOE deficiency is associated with an enhanced susceptibility for the development of non-alcoholic fatty liver disease [28]. However, in agreement with the outcome of our biochemical analyses, sections from the livers of the T0901317-treated mice visually appeared to be full of lipids. They contained medium-sized as well as large lipid deposits that stained relatively more orange than red using Oil red O (reminiscent of the higher triglyceride over cholesteryl ester lipid ratio).

**Figure 2:**
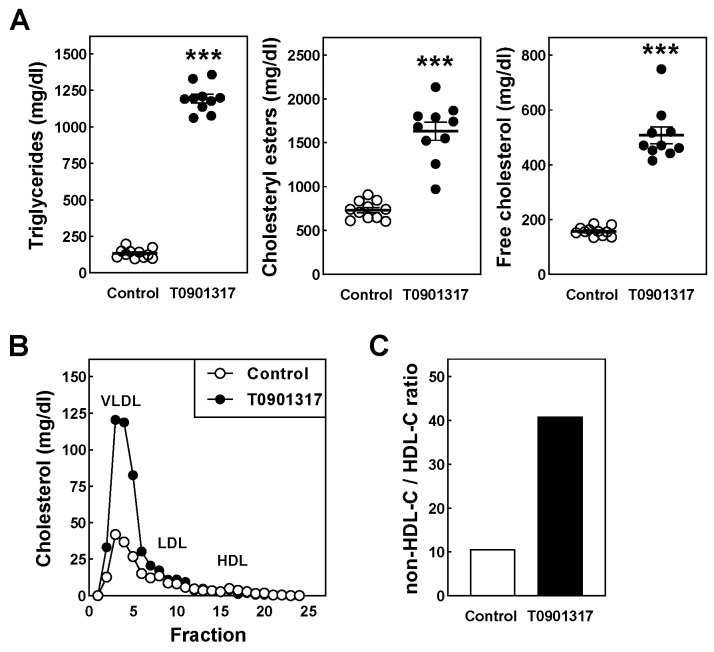
Plasma lipid levels (**A**), the distribution of cholesterol over the different lipoprotein fractions (**B**), and the plasma atherogenic index (**C**) in male *APOE knockout mice* fed a control chow diet (white dots/bars) or chow diet supplemented with 10 mg/kg/day LXR agonist T0901317 (black dots/bars) for 8 weeks. Data represent the individual mouse values with the group means ± SEM (**A**) or single values of pooled plasma of 11 mice per group (**B**,**C**). *** *p* < 0.001 versus control. VLDL, very-low-density lipoprotein (fractions 1–6); LDL, low-density lipoprotein (fractions 7–14); HDL, high-density lipoproteins (fractions 15–21).

The levels of cholesterol and triglycerides were quantified in plasma specimens from the different experimental groups to verify our assumption that the LXR agonism-driven stimulation of hepatic lipogenesis will greatly impact plasma lipid levels under low-fat diet feeding conditions. As can be seen in Figure 2A, the plasma triglyceride levels were 8.9-fold higher (*p* < 0.001) in the T0901317-treated mice as compared to the regular chow-diet-fed controls. In addition, a 2.5-fold to 3-fold increase (*p* < 0.001) in the plasma free and total cholesterol was measured after T0901317 treatment (Figure 2A). A lipoprotein distribution analysis on the pooled plasma revealed that the increase in the total cholesterol levels could be attributed to a 2.9-fold and 1.3-fold rise in the amount of cholesterol associated with pro-atherogenic very-low-density lipoproteins (VLDL) and low-density lipoproteins (LDL), respectively (Figure 2B). The levels of cholesterol associated with anti-atherogenic high-density lipoproteins (HDL) were reduced by 37% by the T0901317 treatment (Figure 2B). As a result, the plasma non-HDL-cholesterol/HDL-cholesterol ratio—a surrogate atherogenic index—was markedly increased from, respectively, 11 in the control mice to 41 in the T0901317-treated mice (Figure 2C).

**Figure 3:**
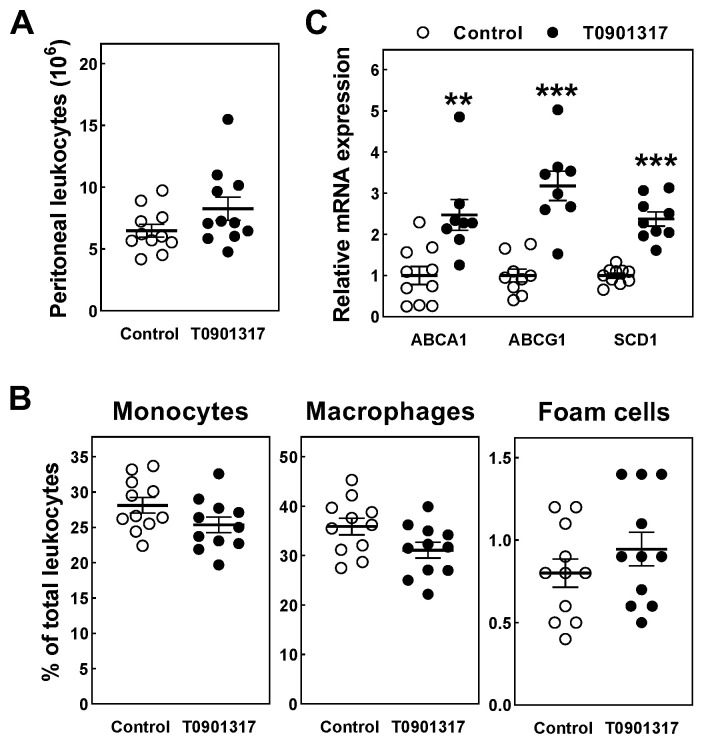
The total number of peritoneal leukocytes (**A**), percentual contributions of monocytes, macrophages and macrophage foam cells to the total peritoneal leukocyte count (**B**), and peritoneal cell relative mRNA expression levels of LXR target genes (**C**) in male *APOE knockout mice* fed a control chow diet (white dots) or chow diet supplemented with 10 mg/kg/day LXR agonist T0901317 (black dots) for 8 weeks. Data represent the individual mouse values with the group means ± SEM. ** *p* < 0.01, *** *p* < 0.001 versus control.

Peritoneal leukocytes were isolated at sacrifice to measure the in vivo macrophage foam cell extent. No difference was observed in the peritoneal total leukocyte count (Figure 3A) or relative monocyte and macrophage numbers (Figure 3B). In agreement with the assumption that LXR agonism initiates a transcriptional response to increase macrophage cholesterol efflux, the peritoneal leukocyte relative mRNA expression levels of both ABCA1 (2.5-fold; *p* < 0.01) and ABCG1 (3.2-fold; *p* < 0.001) were significantly higher in the T090131-treated mice (Figure 3C). In further support of functional LXR agonism in the T0901317-treated mice, a similar 2.4-fold increase (*p* < 0.001; Figure 3C) was detected in the peritoneal leukocyte relative mRNA expression levels of stearoyl-CoA desaturase 1 (SCD1), another established LXR target gene. Importantly, the significant increase in cholesterol efflux-related transcript levels did not translate into a concomitant reduction in macrophage foam cell numbers. As can be appreciated from Figure 3B, the relative number of heavily lipid-laden peritoneal cells was identical in the two experimental groups.

**Figure 4:**
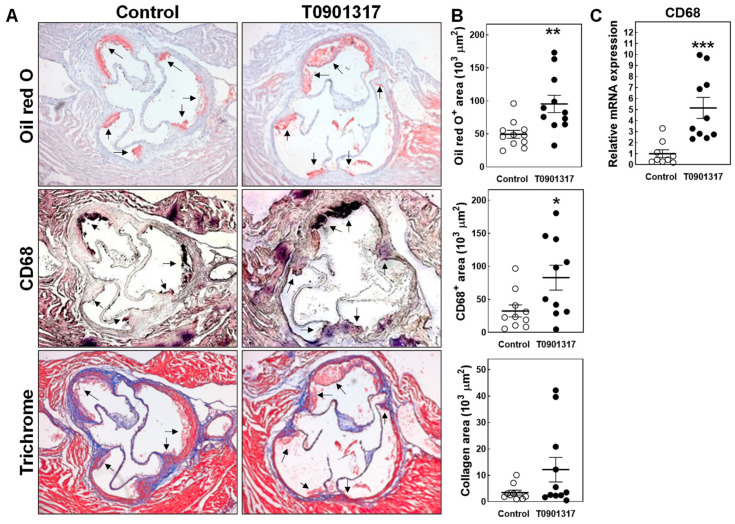
(**A**) Representative images of Oil red O-stained, anti-CD68 antibody-stained, and Masson’s Trichrome-stained cryosections of the artic root from *APOE knockout mice* fed a control chow diet or chow diet supplemented with 10 mg/kg/day LXR agonist T0901317 for 8 weeks; original magnification: 50×. Arrows point towards atherosclerotic lesions. (**B**) Aortic root Oil red O^+^ atherosclerotic lesion sizes, CD68^+^ macrophage areas, and plaque collagen contents, and (**C**) aortic arch relative expression levels of the macrophage marker CD68 in male *APOE knockout mice* fed a control chow diet (white dots) or chow diet supplemented with 10 mg/kg/day LXR agonist T0901317 (black dots) for 8 weeks. Data represent the individual mouse values with the group means ± SEM. * *p* < 0.05, ** *p* < 0.01, *** *p* < 0.001 versus control.

Sections of the aortic root were stained with Oil red O to identify the neutral lipid deposits present in the atherosclerotic lesions (Figure 4A). Quantification of the total Oil red O^+^ plaque area revealed that the atherosclerotic lesions were, on average, 93% larger (*p* < 0.01) after T0901317 treatment. More specifically, the T0901317-treated mice exhibited Oil red O^+^ aortic root atherosclerotic lesions of 95 ± 13 × 10^3^ µm^2^ versus 50 ± 6 × 10^3^ µm^2^ in control chow-diet-fed mice (Figure 4B). As can be appreciated from the representative images in Figure 4A, the lesions in both groups of mice consisted primarily of CD68^+^ macrophage foam cells. As such, T0901317 treatment was also associated with a significant increase in the aortic root CD68^+^ macrophage area (+155%; *p* < 0.05; Figure 4B). In line with the relatively early-stage lesion development, Masson’s Trichrome staining showed that the aortic root atherosclerotic lesions contained limited amounts of collagen (Figure 4A,B). The relative lesional collagen contents were, respectively, 7 ± 1% for the control mice and 10 ± 3% for the T0901317-treated mice (*p* > 0.05). To verify that the atherosclerosis-stimulating effect associated with T0901317 treatment was not dependent on a specific anatomical location, we also quantified the gene expression levels of the macrophage marker CD68 within the aortic arches of the different mice as surrogate measure for the aortic atherosclerotic lesion burden. In agreement with an atherosclerosis-inducing action of T090317, the aortic arch CD68 mRNA expression levels were >5-fold higher (*p* < 0.001) in the T0901317-treated mice as compared to the controls (Figure 4C).

**Figure 5:**
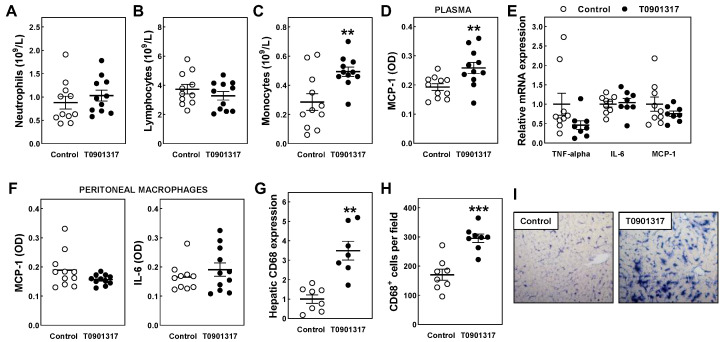
Blood neutrophil (**A**), lymphocyte (**B**), and monocyte (**C**) concentrations, plasma MCP-1 levels (**D**), peritoneal leukocyte cytokine gene expression (**E**) and protein section (**F**) levels, hepatic CD68 relative mRNA expression levels (**G**), and liver CD68^+^ macrophage contents (**H**) in male *APOE knockout mice* fed a control chow diet (white dots) or chow diet supplemented with 10 mg/kg/day LXR agonist T0901317 (black dots) for 8 weeks. Data represent the individual mouse values with the group means ± SEM. ** *p* < 0.01, *** *p* < 0.001 versus control. Panel (**I**) shows representative images of liver paraffin sections that were stained immunohistochemically for the presence of CD68^+^ macrophages; Original magnification: 200×.

The pathogenesis of atherosclerosis is characterized by an interplay between lipids and a variety of immune cells, i.e., monocytes, macrophages, and lymphocytes, that infiltrate plaques from the blood circulation [12]. Given that the increased atherosclerosis burden in the T0901317-treated mice could not be explained by a higher foam cell susceptibility, we investigated whether chronic LXR agonism possibly impacted the blood leukocyte profile and (systemic) inflammatory status. A routine hematological analysis did not uncover a significant difference in the blood concentrations of neutrophils or lymphocytes between the T0901317-treated mice and controls (Figure 5A,B). However, T091317 treatment was associated with markedly increased blood monocyte counts (+73%; *p* < 0.01; Figure 5C). As can be seen in Figure 5D, the monocytosis in the T0901317-treated mice coincided with relatively high plasma levels of MCP-1 (+34%; *p* < 0.01). Although we did observe the anticipated T0901317-induced decrease in relative mRNA expression levels of the pro-inflammatory cytokine TNF-alpha (−54%; *p* = 0.05) [29], no parallel change in IL-6 or MCP-1 gene expression levels was detected within the peritoneal leukocyte fractions (Figure 5E). In accordance, the amounts of IL-6 and MCP-1 protein secreted by peritoneal macrophages ex vivo were not affected by chronic in vivo T090317 exposure (Figure 5F). The increase in plasma MCP-1 levels in response to the T0901317 treatment can, thus, likely not be attributed to a higher rate of cytokine secretion by (tissue) macrophages. Recent studies by Queck et al. suggested that systemic MCP-1 levels are mainly derived from macrophages within the liver and that chronic hepatic lipid accumulation, i.e., as present in non-alcoholic fatty liver disease, is associated with higher plasma MCP-1 levels [30]. In accordance with the notion that T0901317 treatment increased the absolute number (rather than the activity) of macrophages within the liver, the hepatic mRNA expression levels of the macrophage marker CD68 were increased 3.5-fold (*p* < 0.01) in response to T0901317 treatment (Figure 5G). A histochemical analysis of hepatic cryosections validated the presence of a relatively high number of CD68-positive macrophages in the livers of the T0901317-treated mice (Figure 5H,I).

Based upon the findings from our *mice* chronically fed with the chow diet supplemented with T0901317, we hypothesize that the increased atherosclerosis susceptibility was secondary to a pro-inflammatory/pro-atherogenic phenotype of the liver that was generated in response to the initial lipogenic effect of T0901317 LXR agonism in hepatocytes. Previous studies by Grefhorst et al. in C57BL/6 (normolipidemic) *wild-type mice* showed that combined chow diet feeding and oral gavage with 10 mg/kg/day T0901317 for only 4 days was already sufficient to effectively stimulate the lipogenic program and induce hepatomegaly and the accumulation of triglycerides in the liver [4]. To provide support for our working hypothesis that T0901317-induced hepatic lipid accumulation precedes the increase in liver macrophage content, additional groups of male *APOE knockout mice* were fed with the chow diet supplemented with or without 10 mg/kg/day T0901317 for 4 days.

**Figure 6:**
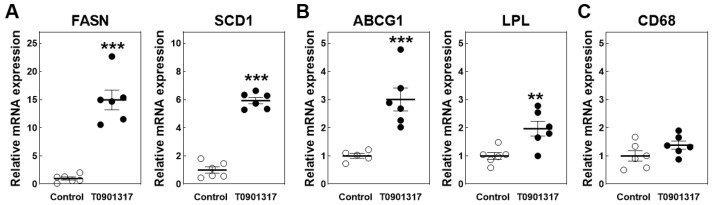
Hepatic relative mRNA expression levels of hepatocyte-associated lipogenic LXR targets (**A**), macrophage-specific LXR target genes (**B**), and the macrophage marker CD68 (**C**) in male *APOE knockout mice* fed a control chow diet (white dots) or chow diet supplemented with 10 mg/kg/day LXR agonist T0901317 (black dots) for 4 days. Data represent the individual mouse values with the group means ± SEM. ** *p* < 0.01, *** *p* < 0.001 versus control.

T0901317 treatment for four days was associated with 15-fold and 6-fold increases (*p* < 0.001 for both) in the hepatic relative mRNA expression levels of the lipogenic LXR target genes fatty acid synthase (FASN) and SCD1 (Figure 6A). The short-term exposure of *APOE knockout mice* to T0901317 also significantly stimulated the liver gene expression levels of the macrophage-associated LXR target genes ABCG1 (3-fold; *p* < 0.001) and lipoprotein lipase (LPL; 2-fold; *p* < 0.01) (Figure 6B). Importantly, the relative mRNA expression levels of CD68 were not significantly different between the livers of the T0901317-treated *mice* and regular chow-diet-fed controls (Figure 6C). It thus appears that the relatively short duration of the lipid accumulation did not yet induce the influx of new macrophages that can produce MCP-1.

## 4. Discussion

In the current study, we evaluated whether chronic LXR agonism remains atheroprotective under low-fat diet feeding conditions. We observed that T0901317 treatment not only failed to reduce atherosclerosis burden, but actually stimulated the development of atherosclerotic lesions in chow-diet-fed hypercholesterolemic *APOE knockout mice.*

In contrast to the significant foam-cell-lowering effect of T0901317 previously detected by Chen et al. in peritoneal macrophages of high-fat-diet-fed *APOE knockout mice* [19], T0901317 treatment did not change the peritoneal macrophage foam cell extent in our chow-diet-fed *APOE knockout mice*. Based upon these combined findings, one could suggest that the ability of LXR agonism to reduce the macrophage foam cell extent is diet-dependent. However, it should be noted that the plasma cholesterol levels were not different after T0901317 treatment in the studies by Chen et al., whilst a marked ~4-fold increase in the plasma atherogenic index (aggravated hypercholesterolemia) was seen in the T0901317-treated *mice* as compared to the controls in the current study. In this light, our findings provide additional support for the generally accepted notion—originally derived from studies by Venkateswaran et al. [31]—that a rise in cholesterol efflux capacity due to an LXR agonism-associated increase in ABC transporter transcription enables macrophages to effectively deal with a potentially foam-cell-inducing rise in plasma cholesterol levels. Furthermore, these data concur with the clinical observation of Rohatgi et al., that a higher ability of plasma high-density lipoproteins to remove cholesterol from macrophages is independently associated with a reduced atherosclerotic cardiovascular disease risk [32].

Given that the increase in atherosclerosis susceptibility was not due to an accelerated generation of macrophage foam cells, changes in other (pathological) processes probably underlie the relatively high plaque burden detected in the T0901317-treated *mice*. The interaction of MCP-1 with CC chemokine receptor 2 (CCR2) drove the migration of monocytes from the bone marrow into the blood and to sites of inflammation, i.e., atherosclerotic plaques. Boring et al. [33] and Dawson et al. [34] found that genetic CCR2 deficiency in chow-diet-fed *APOE knockout mice* protected against the development of atherosclerosis. In this light, it is anticipated that the increase in plasma MCP-1 levels and associated monocytosis contributed significantly to the T0901317-induced increase in atherosclerosis susceptibility. Alisi et al. observed that circulating MCP-1 levels were higher in children with (biopsy-proven) non-alcoholic fatty liver disease than in age-matched obese controls and that these levels positively correlated with disease severity [35]. Queck et al. detected a similar positive association between blood MCP-1 levels and liver disease degree (fibrosis score) [30]. The fact that chronic treatment with T0901317 increased liver triglyceride levels and macrophage content may, thus, explain the observed increase in plasma MCP-1 levels and the associated rise in blood monocyte levels and atherosclerosis susceptibility. In agreement with a pro-atherogenic effect of fat accumulation within the liver also in the human clinical setting, Jin et al. recently described that the non-alcoholic fatty liver disease fibrosis score significantly correlates with coronary artery disease severity and is independently predictive of cardiovascular events and mortality in patients with stable atherosclerotic coronary artery disease [36].

## 5. Conclusions

We showed that the treatment of chow-diet-fed male *APOE knockout mice* with a standard oral dose of 10 mg/kg/day LXR agonist T0901317 was associated with hepatic fat accumulation, exacerbated hyperlipidemia, and monocytosis and stimulated early atherosclerotic lesion development. These findings highlight that the pro-atherogenic hepatic effects of LXR agonism are dominant over the anti-atherogenic effects in macrophages in determining the overall atherosclerosis outcome under low-fat diet feeding conditions. A low-fat diet experimental setting, as compared to the commonly used high-fat-diet-based preclinical setup, thus appears more sensitive in uncovering the potential relevance of the off-target liver effects of novel anti-atherogenic therapeutic approaches that target macrophage LXR.

## 6. Limitations and Future Directions

We showed that T0901317 treatment worsens atherosclerotic lesion outcome, i.e., accelerates plaque development, in *mice* that contain early-stage lesions. However, this observation does not necessarily imply that a similar effect of LXR agonism would be detected in more advanced lesions that are generally present in human subjects suffering from cardiovascular disease. Therefore, our current findings should be followed up with studies on the impact of LXR agonist treatment on the progression and/or regression of established, more inflammatory, and necrotic atherosclerotic lesions. Replication of our experiment in *mice* with a higher age at the start of the study may also be required, since Gogulamudi et al. [37] showed that age can significantly affect the atherogenic diet-feeding-associated hypercholesterolemia extent and lesion development outcome in *APOE knockout mice*. Furthermore, although we do not anticipate that the gender of the *mice* will significantly affect the overall impact of T0901317′s actions, it is recommended to perform future studies in both male and female *mice*. 

## Data Availability

All data associated with the study that are not already presented in the paper can be obtained from the corresponding author upon reasonable request.
